# Supplementary material for: Causal Relationship between Aging and Anorexia Nervosa: A White-Matter-Microstructure-Mediated Mendelian Randomization Analysis
Source: Biomedicines. 2024 Aug 16;12(8):1874. doi: 10.3390/biomedicines12081874 (PMC11351342; doi:10.3390/biomedicines12081874)
Supplement: Supplementary file 1 [file biomedicines-12-01874-s001.zip › Supplementary Figure/Figure S9/Leaveoneout plot of MR analysis for ubm-b-1906 to AN.pdf]

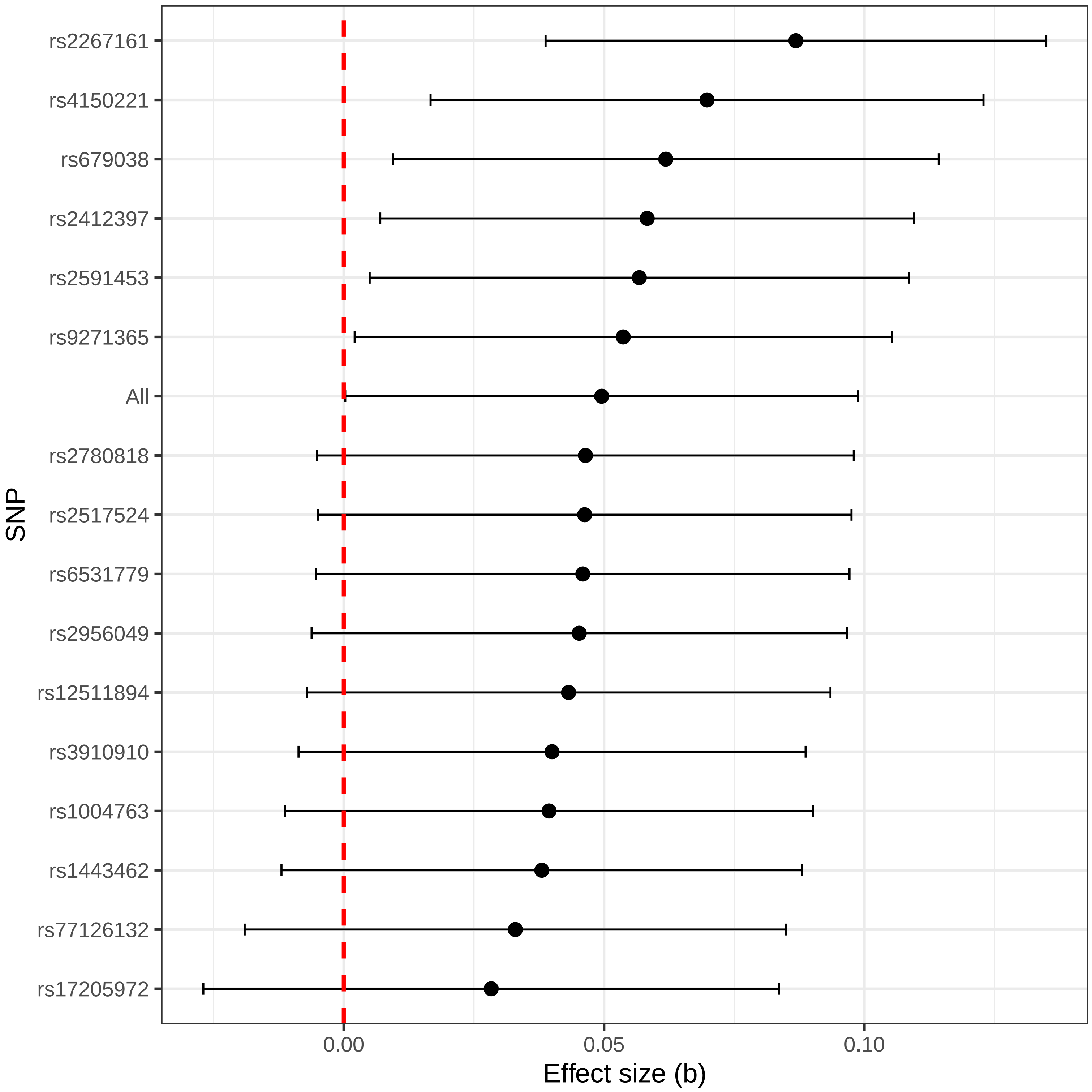
rs2267161
rs4150221
rs679038
rs2412397
rs2591453
rs9271365
All
rs2780818
rs2517524
rs6531779
rs2956049
rs12511894
rs3910910
rs1004763
rs1443462
rs77126132
rs17205972
SNP
0.00
0.05
0.10
Effect size (b)
